# An Innovative Approach in Rural Aging Education: Extension’s Impact Through Healthy Aging Webinars

**DOI:** 10.1093/geroni/igaf122.1631

**Published:** 2025-12-31

**Authors:** Christine Fruhauf, Natalie Bachmeier, Ginger Williams, Sue Schneider

**Affiliations:** Colorado State University, Fort Collins, Colorado, United States; Colorado State University, Fort Collins, Colorado, United States; Colorado State University, Fort Collins, Colorado, United States; Colorado State University, Fort Collins, Colorado, United States

## Abstract

While rural communities possess unique strengths, limited access to aging services can detrimentally impact individuals’ health-related outcomes. Colorado State University Extension focused its engaged education on innovative approaches to Health Extension and learned that county-level Extension specialists need additional training to effectively support older adults and improve services in rural communities. Thus, in this presentation we will describe how we increased the confidence of interdisciplinary Extension specialists and community partners to respond to aging-related needs in rural communities through our Advancing Healthy Aging (AHA) webinar series. Webinar topics were identified during a needs assessment and delivered in fall 2024 and spring 2025. Of the eight webinars, to date, a total of five AHA webinars were delivered online with 193 attendees. Highest attended webinars addressed reducing ageism (n = 60), reducing social isolation and increasing belonging (n = 43), and avoiding caregiver burnout (n = 36). A total of 75 attendees completed an evaluation administered after each webinar. Data indicate that 93% (n = 70) of respondents reported an increase in knowledge and ability to apply knowledge gained. Additionally, 95% (n = 71) of respondents reported increased confidence in educating people about healthy aging and in directing people to additional resources; comments gathered from open-ended questions support the findings. Limitations include more than half (61%) of attendees did not complete the evaluation and may have different views on the webinars’ impact. Despite limitations, the AHA webinars have potential to further impact rural communities as they were recorded and posted publicly.

